# The physiological and perceptual effects of plant extracts (Catha Edulis Forsk) during sustained exercise

**DOI:** 10.1186/s13011-016-0063-4

**Published:** 2016-05-10

**Authors:** Mowaffaq Awad Sallam, Kamaludin Ahmed Sheikh, Ronald Baxendale, Mohammad Nurul Azam, Maged El-Setouhy

**Affiliations:** Substance Abuse Research Center, Jazan University, Jazan, Kingdom of Saudi Arabia; School of Life Science, College of Medical, Veterinary and Life Science, University of Glasgow, Glasgow, UK; Researchers support and service unit, Deanship of Scientific Research, King Saud University, Riyadh, Kingdom of Saudi Arabia

**Keywords:** Khat chewing, Physiological effects, Perceptual effects, World Anti-Doping Agency

## Abstract

**Background:**

Khat (Catha Edulis Forsk) is a natural psychoactive substance that contains addictive substances such as Cathine and Cathinone which have similar structure and action to amphetamine. This substance has been suggested that it can decrease perceived exertion and thus improve performance. There is no study in the literature regarding the effect of khat on exercise performance. Therefore, the aim of this study is to find out whether khat leaves can decrease perceived exertion in humans.

**Methods:**

This study is an experimental crossover study conducted at the Substance Abuse Research Centre in Jazan University, Saudi Arabia. Twenty one healthy volunteers were randomly assigned into two experiment trials. Each volunteer visited the lab three times. The first visit was a familiarization session about the nature of the study and the equipment. On the second visit, 45 min before the experiment volunteers ingested either 33 ml of fruit juice (placebo) or the juice mixed with 45 g of ground khat leaves. Then the participants were instructed to perform a 10 Km cycling on an ergometer and recorded the following physiological variables repeatedly on every 5 min of cycling: heart rate, time to complete 10 km cycling, tympanic temperature, and perceived exertion rate. On the third visit a crossover trial was conducted one week after the second visit; then the same cycling test was performed and the same variables were recorded as the second visit. The experimental protocol was reviewed and approved by Research Ethical Committee of the Medical Research Centre, Jazan University.

**Results:**

According to study results, khat dramatically decreased time taken to complete a 10 km cycling time trail (*p* < 0.05), and significantly increased heart rate (*p* < 0.05) and tympanic temperature (*p* < 0.01). However, khat did not reduce participant's perceived exertion during the physical trial. The Bonferrini simultaneous confidence intervals using multivariate Hotelling’s T^2^ was performed to test the significance of the mean vectors for the placebo group and the Khat group and found that groups are statistically significant.

**Conclusions:**

Khat showed a clear enhancing effect on physical performance. The most parsimonious explanation for this effect is that, like the related amphetamines, cathine/cathinone act as stimulants to increase the capacity to perform exercise. Thus, khat produces the same effects which lead to the banning of amphetamine. These findings conform & endorse the recent prohibition of cathinone by the World Anti-Doping Agency (WADA, 2014).

## Background

The use of stimulants for endurance in exercise-based sports is not a new phenomenon; it dates back to the times of ancient Greek Olympic Games (776 BC). Stimulants are used to enhance performance and to delay fatigue [1]. Amphetamine, cocaine and caffeine are the most commonly used stimulants in sport [2]. Khat (Catha edulis Forsk) is a mild stimulant that shares a pharmacological and structural similarity to amphetamine [3]. Khat is commonly believed to improve work capacity and increase energy levels and alertness [4]. Khat is often consumed by people employed in exhausting jobs, such as construction workers and long distance drivers, and by students preparing for examinations [4–7]. Its use is perceived to improve concentration and counteract fatigue [4–7]. It is reported that Khat gives the users feelings of euphoria and stimulation [8, 9].

Khat chewing is a very popular and deeply-rooted socio-cultural tradition in regions of East Africa and the Arabian Peninsula. This is especially well developed in Yemen where it is used as a recreational drug even at social events like weddings and meetings called “khat sessions” [10]. Khat is chewed like tobacco and kept for a while in the cheek; to suck and swallow its juice, along with some amount of water to counter the bitter taste of the plant [10]. During a typical event, a participant will chew 100–200 g of khat [11]. It is estimated that there are now five to ten million regular khat users in the world, with khat users spending 6 to 20 US$ per day on its use [3]. The main active ingredients of khat leaves are cathinone, cathine and Norephedrine. Cathinone and cathine are similar to amphetamine in structure, effects and pharmacological activity but with lower potency [11–16]. Cathinone is more potent than cathine, and they are listed as Schedule I and IV drugs, respectively [11, 13, 17–19]. The use of cathine (nor-pseudoephedrine) is prohibited if its concentration in the urine exceeds 5 micrograms per millilitre [20]. On September 11 2014, the World Anti-Doping Agency (WADA) had added cathinone on the List of Prohibited Substances [21].

Despite the clear stimulant actions of khat, and historical use of stimulants in physically demanding sports, there has been a lack of studies investigating the effects of khat on physiological functions of the body, such as endurance. The main objective of this study was to investigate the physiological effect of khat and perceived exertion during strenuous exercise. Our approach compared the effects of khat and placebo on resting heart rate, tympanic temperature, time taken to complete a standardized 10 km cycle track, and rating of perceived exertion.

## Methods

### Study participants

The study was carried out among young healthy human volunteers. They were mostly students at Jizan University and members of a local football team. Some of the volunteers were not students. The volunteers were regular and irregular Khat users and in a good health. This was confirmed by the following inclusion criteria: participants should not have any heart problem, no chest pain when doing activities, not currently taking any medication for a health problem. In total, 21 volunteers were recruited. Their average age was 23.4 years ± 2.2 years, and average weight was 66.8 kg ± 5.6 kg. All participants completed all trails. There were no drop outs or injuries.

### Design and procedures

This study is an experimental crossover study conducted at the Substance Abuse Research Centre in Jazan University, Saudi Arabia. Tests started in early December 2011 and finished about 3 months later. All participants completed two trials one after consuming a placebo and the other after consumption of Khat. The order in which the tests were conducted was randomized. The participants were blinded to the nature of the test. The author was not blinded because he had to make up the drinks. Since, the results were recorded by machine we hope the experimenter effects and biases are very minimal. A statistical power test on pilot data suggested that the number of subjects required to carry out this experiment is 21 subjects. The sample size calculated was based on standard deviation of 1.0, expected effect size of 0.643 (Cohen’s d, a measure of effect size), power level of 0.80 and alpha of 0.05 [22].

Each volunteer visited the lab three times (baseline/familiarization and the two test days). On the first visit a familiarization session was conducted to make volunteers become familiar with the nature of the study and the equipment, then consent to proceed was sought and a simple health questionnaire was completed. On the second visit, 45 min before the experiment volunteers ingested either 33 ml of fruit juice (placebo) or the juice mixed with 45 g of ground khat leaves which had been filtered to remove particles — on average 100 g of khat leaves there are 114 mg cathinone, 83 mg cathine and 44 mg norephedrine [10]. The time interval period of 1 week was chosen to avoid fatigue effects and loss of concentration. Previous studies indicate that this time period allows adequate time for absorption of khat [23]. Also, the maximal concentration effect of cathinone is expected 15–45 min after an oral ingestion [24].

**Fig. 1:**
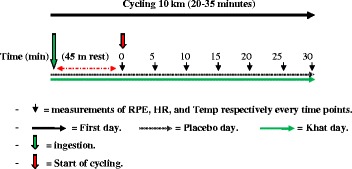
The experimental protocol of the study

After ingestion of the drink, participants were instructed to perform a 10 Km cycling on a RacerMate CompuTrainer cycle ergometer and recorded the following physiological variables repeatedly on every 5 min of cycling: heart rate, time to complete 10 km cycling, tympanic temperature, and perceived exertion rate. On the third visit (the crossover session) the participants returned to the lab after 7 days at the same time of last tests and each participant ingested the other drink given in the second session; then the cycling test was performed and the same variables in the second session were recorded. The 1 week time interval was chosen to avoid fatigue effects and loss of concentration. Previous studies show this allows adequate time for absorption of khat. (See Fig. 1).

### Instruments and measurements

There were two CompuTrainer sets in the lab. During the cycling trials some of the riders were tested in pairs to allow for some sense of competition while others were tested in isolation and the essence is that this study is not two man race assessments. All 21 riders were tested in both trials (khat and placebo). All pairs of experiments were conducted in the similar conditions. The laboratory was air conditioned and the temperature was maintained at 21 °C. Some volunteers preferred to exercise with the laboratory windows opened. The experiments were conducted during the winter in Jizan when the outside temperature could be about 30 °C.

#### 10 Km cycle tests

**Fig. 2:**
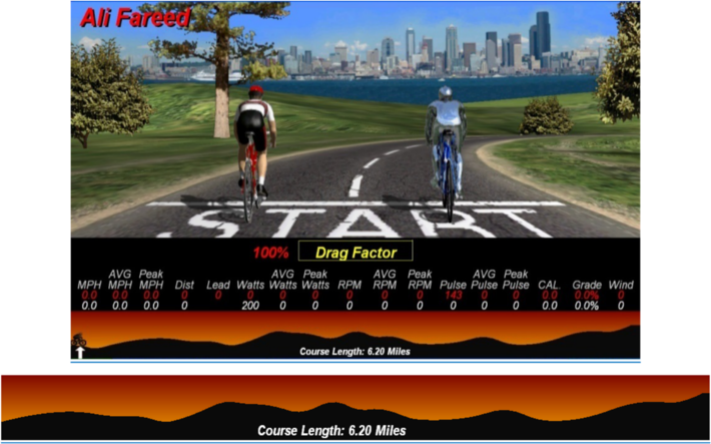
Shows the CompuTrainer_TimeTrail.3 dc course and the distance of this course

The test used a RacerMate CompuTrainer cycle ergometer to provide a simulation of a 10 km time trial (http://www.racermateinc.com/computrainer/). The ergometer is controlled by a laptop computer and the software delivers specific load to replicate ascents, and descents. The CompuTrainer_TimeTrail.3 dc course was used for tests. The distance of this course is 10 km and it contains mix of climbs and descents. The Computrainer screen simulates the participants during the experiment, where the screen shows the performance of the competitors during the race time. The Computrainer screen is shown in Fig. 2.

#### Heart rate

**Fig. 3:**
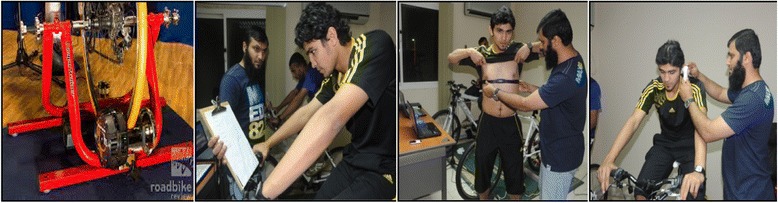
This shows details of the experimental measurement of time trial, perceived exertion, heart rate and core temperature. The computrainer device is shown in the left panel. Details of the rate of perceived exertion scale (Borg’s RPE 20-point Scale) and Polar Heart Rate Monitor are in the middle panel. The right panel shows a volunteer whilst their temperature is measured

Heart Rate was measured continuously during cycling by Polar FT1 Heart Rate Monitor Watch, U.K (http://www.fitnessmegastore.co.uk/heart-rate-monitors-87.html). It is shown in Fig. 3 below. The heart rate was recorded every 5 min during the trial.

#### Temperature

Each participant’s tympanic membrane temperature was recorded using an Omron Digital Ear Thermometer, (mp 510 MC-510-E2, Omron Healthcare, Kyoto, Japan LTD). This was recorded after each heart rate measurement. This procedure is shown above in Fig. 3.

#### Rate of Perceived Exertion (RPE)

The volunteers rating of perceived exertion was measured every 5 min during the time trial with a Borg 20-point scale [25]. The English version of the scale has been translated into Arabic by the staff in King Saud University. The translation procedure involved forward and backward translation from English to Arabic language. The questionnaire was assessed for face and content validity by a group of local experts in the field. Prof Hazza’s group had used this translation successfully for several years. This procedure is shown in Fig. 3.

### Statistical analysis

All data were collated in Microsoft Excel 2010 and the following statistical software were used for data analysis: SPSS version 18 was used for paired-sample *t*-test analysis and repeated measures analysis (ANOVA), Minitab version 16 was used for graphical analysis and finally SAS version 9.3 was used for multivariate analysis. The data were first tested to confirm the assumptions of repeated measures ANOVA such as normality, Sphericity and equality of variances condition. Subsequently, graphical analysis and paired t-tests were used to compare the mean differences of the group; this determines whether there is significant mean difference in heart rate, perceived exertion and temperature scores among participants in the two trials (khat and placebo).

Repeated Measures Analysis of Variance (ANOVA) was utilized to find the effect of group differences in heart rate, perceived exertion and temperature. We tested both the within-subject effects and between-subject effects of the model factors. The experimental design used was essentially a two-factor (time point and treatment) experiment with repeated measures of 5 responses on both factors. In each model, the subject is entered as a random factor while Time Point and Treatment (placebo and khat) and the interaction between Time Point and Treatment were entered as between-subjects factors. The Levene statistic test was used to assess the assumption of equality of variances in the study variable of the two trial groups. The Bonferrini simultaneous confidence intervals using multivariate Hotelling’s T^2^ was performed to test the significance of the mean vectors for the placebo group and the Khat group.

## Ethical approval

This study was conducted in accordance with the ethical guidelines of the Declaration of Helsinki; and it had been approved by Research Ethics Committee (REC) of the Medical Research Centre (MRC), Jazan University (Reference number 110). After approval of the study protocol by the Research Ethics Committee, informed consent was obtained from all participants. Participants were told they could withdraw from the experiment at any time. No volunteers withdrew during the experiment. Volunteers were not paid for participating. Any minor financial costs such as travel expenses were repaid from the project budget. The risks associated to this study were very minimal and provision was made to cover any medical costs associated with the experiments. Some side effects which may occur as a result of taking the khat leaves are: increased heart rate, blood pressure, headaches, restlessness and alertness. The study participants were regular and irregular khat chewers and the given khat dosage (45 g) is quite conservative therefore we do not expect any serious side effects to occur.

## Results

### Time taken to complete 10 km cycle

**Fig. 4:**
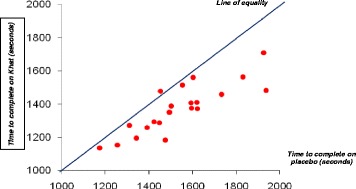
This figure plots the time to complete the 10 km time trial after consuming khat or placebo for all 21 volunteers. Only one person took longer to complete the 10 km cycle when using khat while most volunteers finished about 2–3 min faster when using khat. To find out if there is a significant change in the time required to complete the 10 km cycle between using the placebo and khat, the differences in time taken were calculated

The time taken to complete the time trial was reduced after taking khat when compared to placebo for each participant (Fig. 4). Only one person cycled the route slower after consumption of khat. The other 20 volunteers were faster. The difference in times in the two conditions appears to be smaller for the faster cyclists, say those who took less than 1600 s, than it is for the slower cyclists. The minimum time required for cycling 10 km is not much different (0.4 %) between placebo and khat. The maximum times were more different (4.7 %), 34 min 17 s after placebo and 28 min 30 s after consuming khat (See Fig. 4).

On average, after consuming khat the trial was completed 168.1 s faster than when using the placebo. The mean time after placebo was 1554 ± 230 s. The mean time after consuming khat was 1386.2 ± 156 s. When these times were compared using a paired *t*-test the result was highly significant (t = 5.88, df = 20, *p* < 0.001). The magnitude of the change was not uniform across the group of volunteers. It is easy to see in Fig. 4 that the improvement in the faster cyclists is relatively uniform. Those who complete the placebo trial in 1600 s or less improve by about 10 % after consumption of khat. The five slowest cyclists make much larger improvements of about 15 to 25 %. This differential effect makes the analysis more complicated because the assumptions of the General Linear Model are not secured.

**Fig. 5:**
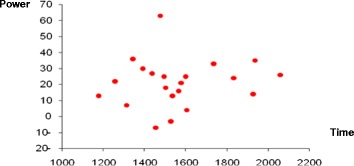
This shows the difference in mean power (khat minus placebo) against the time taken to complete the trial on placebo for each participant. The zero line shows no difference between the two trials. Points above the line indicate increases in power. Points below the line show decreases

The software recorded the mean power output of each volunteer over the time trial. 19 of the 21 volunteers increased their power after consumption of khat. The mean increase was 21 watts. The range was from increase of 61 watts to a decrease of 7 watts. A paired *t*-test showed that the mean power after consumption of khat was significantly higher (t = 7.12, df = 20, *p* < 0.001) (See Fig. 5).

### Physiological measures and Khat consumption

#### Heart rate

**Fig. 6:**
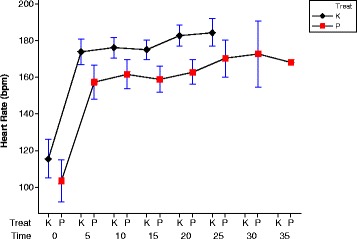
This show the mean heart rate and SEM after taking the placebo (P) and khat (K). There is a clear increase on the means of heart rate in both khat day and the placebo day during 5 min after starting then steady. The heart rate after consumption khat is higher at all-time points comparing with taking placebo. Red symbols show data from the placebo day and the black symbols from the khat day. The last point on red symbols appears no bar because there is only one person took longer time to complete the trial course

In both placebo and khat groups, heart rate rises during the first 5 min of exercise and then is stable during the exercise (See Fig. 6). At all time-points, the mean heart rate was increased in khat consumption group: khat group heart rate increase was 12.1 BPM at t = 0 and 19.9 BPM at t = 20. Comparison of mean heart rate toward the end of the trial shows this increase in heart rate is significant (t = 8.077, df = 20, *p* < 0.001; 15.8 + 11.5 bpm higher). Note that the time trial is completed earlier after consumption of khat.

#### Temperature

**Fig. 7:**
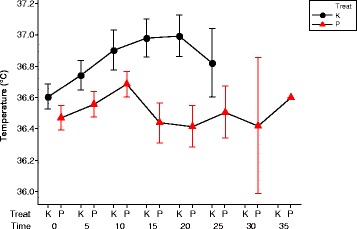
This shows the mean and SEM of temperature for all volunteers after taking either the placebo (P) or khat (K). Red symbols indicate the placebo day and black the khat day. The temperature rises after khat consumption and falls later after 20 min. While temperature rises for 10 min after consuming the placebo then it becomes unstable

Tympanic temperature appears higher after khat ingestion, but this is only significant later in the trial (See Fig. 7). There was no significant difference between the mean temperature of the placebo and khat groups early in the trial: at time 0 [t = 1.550, df = 20, *p* > 0.05], at time 5 [t = 1.420, df = 20, *p* > 0.05], or at time 10 [t = 1.540, df = 20, *p* > 0.05], but later in the trial the increase in temperature of the khat group become significant: at time 15 [t = 4.038, df = 20, *p* < 0.001] and at time 20 [t = 3.452, df = 20, *p* < 0.001].

#### Perceived exertion

**Fig. 8:**
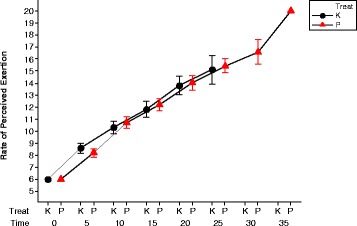
This shows the mean (and SEM) of rate of perceived exertion of all volunteers after consuming khat and after taking the placebo. Red symbols show data from the placebo day and the black symbols show data from the khat day. There are increases in the both means of perceived exertion during the khat and placebo trials

The mean RPE scores for both time trials are plotted in Fig. 8. It is clear that the scores are very similar at the same time points in both trials up to 20 min. That means, the mean perceived exertion at the five time trials in both groups (khat and placebo) are not significantly different from one another at time point 5 (t = 0.940, df = 20 *p* = 0.358); at time point 10 (t = 0.779, df = 20 *p* = 0.445); at time point 15 (t = 0.574, df = 20 *p* = 0.572) and at time point 20 (t = 0.748, df = 20 *p* = 0.748).

#### Measures of physiology and perceived exertion

**Fig. 9:**
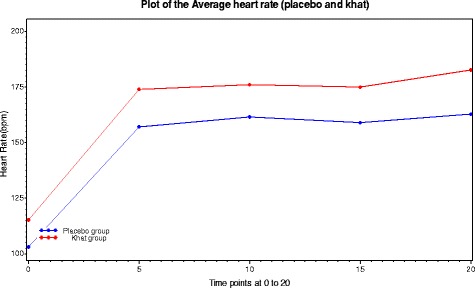
The plot of the Average heart rate of Plesbo and Khat using Bonferroni simultaneous confidence intervals

The Hotelling’s T^2^ value is 26.14 and F = 4.705 with degrees of freedom 5 and 36. The p-value for the test is 0.00208 and we conclude that the mean vectors for the placebo group and the Khat group are statistically significant. This multivariate test takes into account the positive correlation between the two measurements for each group – information that is unfortunately ignored by the univariate tests (See Table 1 and Fig. 9).

**Table 1:** Multivariate calculations for Measures of physiology and perceived exertion

Time	Placebo Mean(sd) (*n* = 21)	Khat Mean(sd) (*n* = 21)	95 % Confidence Interval	
			Bonferroni	Simultaneous
0	103.2 (658.3)	115.3 (539.8)	−40.1–15.9	−32.5–8.3
5	157.2 (414.5)	173.9 (234.6)	−37.3–3.9	−31.7– −1.7
10	161.7 (305.3)	176.1 (151.3)	−31.7–2.9	−27.0– −1.8
15	158.9 (250.1)	175.0 (134.7)	−32.0– −0.2	−27.7– −4.5
20	162.8 218.0)	182.7 (154.7)	−35.5– −4.3	−31.3– −8.5

### Repeated measures analysis (ANOVA)

The Mauchly’s test indicated that the assumption of sphericity in the model had been violated (HR *χ*^2^ 71.806, df = 9, *p* < 0.001; for Temp *χ*^2^ 71.806, df = 9, *p* < 0.001 and for RPE *χ*^2^ 71.806, df = 9, *p* < 0.001) that means the covariance matrix assumption was not met, therefore the F-values were corrected using the Greenhouse-Geisser test. The results of the Anova indicated a significant time effect of the Within-Subjects of the following three variables Heart Rate (F = 177.257, df_1_ = 4, df_2_ = 1.412, *p* < 0.01), Temperature (F = 2.511, df_1_ = 4, df_2_ = 2.301, *p* < 0.05), and RPE (F = 152.675, df_1_ = 4, df_2_ = 2.090, *p* < 0.01); that means we reject the null hypothesis of equal means in these variables. The within subject test indicated that there is a significant time effect in the model, that means, the experiment groups (placebo and khat) effects do change over time and will get higher heart rate, higher temperature and higher RPE over time. In addition, the Within-subject test also indicated that there were no significant interaction effects in any of the variables – session and time (HR F = 0.523, df = 1.412, *p* > 0.05; for RPE F = 0.442, df = 2.090, *p* > 0.05; Temp F = 3.157, df = 1, *p* > 0.05). To conserve space, details of the results of the Main effects plot & the interaction plot are available upon request.

The results of Between-Subjects Effects indicated a significant main effect of a session (placebo and khat) in heart rate (F = 15.387, df = 1, *p* < 0.05), and tempreture (F = 9.335, df = 1, *p* < 0.01), while no difference was found between RPE scores in placebo and khat (F = 0.059, df = 1, *p* > 0.05). Finally, the equality of variances condition was fulfilled as the p-values of Levene’s test were greater than 0.05 across all the three variables in the study. We can conclude that the differences between condition Means are likely due to chance and not likely due to khat consumption.

## Discussion

The primary aims of this study was to investigate the effects of khat consumption on physiological factors in a period of self-paced, moderate intensity exercise lasting 20 to 30 min. Permission was obtained to test 21 normal healthy young men. They all completed the experiment without ill effects. After consuming khat the time taken to complete a 10 km cycling time trial was reduced, the mean work rate was increased, the mean heart rate and tympanic temperature were higher; see Fig. 4, Fig. 5, Fig. 6 and Fig. 7.

The main finding in the present study indicated that the time to complete 10 km time trial was reduced from a mean of 1554.3 ± 230.7 s to a mean of 1386.2 ± 156.8 s after consumption of khat (t = 13.169, df = 20, *p* < 0.001). Over the period of the time trial, the mean of heart rate and tympanic temperature were significantly higher ([t = 8.077, df = 20, *p* < 0.001] and [t = 3.452, df = 20, *p* < 0.005] respectively) after khat consumption when compared with the placebo. The specific reason for assessing tympanic pressure is that khat is associated with increase in diastolic blood pressure and cycling for untrained personal usually increases blood pressure [26]. The Bonferrini simultaneous confidence intervals using multivariate Hotelling’s T2 test reveals that the mean vectors for the placebo group and the Khat group are statistically significant (See Fig. 9 & Table 1).

The heart rate was higher and stable throughout the experiment; see Fig. 6. The temperature rises in placebo trial and then returns to normal, see Fig. 7. Khat trial rises temperature for longer to higher peak value then begins to come down, more heat due to produce high work then slowly return to normal. Heat loss as sweat mechanism maybe led to temperature homeostasis after the increase as a response to the feedback afferent from central and peripheral sensors [27, 28]. This result from the present experiment is in accord with many of studies [29–33] which reported in literature review that consumption of khat induces to increase the body temperature after chewing.

The perceived exertion of the volunteers was measured with a Borg scale, after translation to Arabic by the staff in the exercise physiology laboratory in King Saud University in Saudi Arabia. They have considerable experience of using this successfully with Arabic speakers. The effectiveness of the use of Borg scale may differ between volunteers according to their individual evaluation of their perceptions. This may be a small problem since the same volunteers were tested twice and their differences were measured.

The results clearly show that consumption of khat improves cycling time trial performance. The results of this study show that participants worked harder and improve their performance after khat consumption. They had higher heart rates and tympanic temperatures. In addition, they probably have higher oxygen consumptions but no equipment to measure oxygen consumption was available at the time of doing these experiments at SARC. Consumption of khat must have a significant action on the mechanisms responsible for perceiving exercise intensity. To our knowledge, no previous investigation had measured the rate of perceived exertion during a khat trial. The present investigation not only the first to measure the rate of perceived exertion (RPE) during prolonged exercise following khat ingestion, but also the first dosage of khat that have been shown to alter exercise performance.

The participants in this study were all novice cyclists. However, they are all young fit men most of whom engage in regular sporting activities such as playing football, without displaying any particular training history. Using cycling as an exercise modality was necessary there was no treadmill in the SARC centre at the time of experiment. Cycling is not common in Jizan and all the volunteers, except two, were novices. This was offset by allowing time for familiarization with the cycles. The Computrainer ergometer provided consistent tests and allowed the workload to be estimated.

There is an interesting possibility that the effect of khat in improving exercise performance is mediated through the ‘central governor model’ proposed by Noakes rather than through improved cardiovascular performance. The central prediction of the central governor model is that the central nervous system determines the work rate that can be sustained for the anticipated duration of exercise, as well as the moment of exercise terminates. Thus, the brain does not recruit additional motor units during prolonged exercise because that would threaten the capacity to maintain homeostasis which would in turn lead to the termination of exercise, organ damage or failure. As a result, the brain paces the body during exercise to ensure that the activity is completed without any loss of cellular homeostasis [27, 28].

During exercise continuous feedback from many systems in the body will inform the central controller of the state features such as fuel reserves and the rate of heat accumulation state among other variables. This continuous feedback is integrated to regulate the exercise intensity [27, 28, 34, 35]. The most likely action of Khat is to change the relationship between the inputs and outputs so that higher exercise intensities are tolerable. This is seen in the increased tympanic membrane temperature of volunteers, the higher heart rates and higher work rates yet the RPE values lower at the end of the exercise period. These results clearly show that consumption of khat improves cycling performance. The heart rate and core temperature are higher but the perceived exertion is lower.

### Limitations and future research implications

The limitations of the current study include: 1) the study did not compare the perceived exertion of the athletic and non-athletic groups of the study volunteers; 2) some of the volunteers were regular khat users while other irregular khat users therefore we do not know how might this have affected results; and 3) during the cycling trials some of the riders were tested concurrently while others were tested in isolation and the study did not control this factor. Hence, all the research limitations highlighted above needs to be explored further, and could be a focal point for future studies. Finally, we recommend future researchers to investigate the ergogenic effects of khat extract and using variables such as grip strength, reaction time, and blood pressure etc.

## Conclusion

This study is the first to investigate the effects of khat on physiological functions of the body, during strenuous exercise. These results clearly showed that khat consumption leads to higher work rates and improves performance in addition to reductions in the fatigue. It is speculated that cathine/cathinone exert a central action to increase the capacity to perform exercise. Thus, khat produces the same effects which lead to the banning of amphetamine. These findings conform & endorse the recent prohibition of cathinone by the World Anti-Doping Agency (WADA, 2014).
